# Higher Photochemical Quenching and Better Maintenance of Carbon Dioxide Fixation Are Key Traits for Phosphorus Use Efficiency in the Wheat Breeding Line, RAC875

**DOI:** 10.3389/fpls.2021.816211

**Published:** 2022-02-04

**Authors:** Van Lam Nguyen, Lachlan Palmer, James Stangoulis

**Affiliations:** College of Science and Engineering, Flinders University, Bedford Park, SA, Australia

**Keywords:** photosynthesis, gas exchange, chlorophyll fluorescence, phosphorus use efficiency, wheat

## Abstract

Maintaining carbohydrate biosynthesis and C assimilation is critical under phosphorus (P) deficiency as inorganic P (Pi) is essential for ATP synthesis. Low available P in agricultural soils occurs worldwide and fertilizer P sources are being depleted. Thus, identifying biosynthetic traits that are favorable for P use efficiency (PUE) in crops is crucial. This study characterized agronomic traits, gas exchange, and chlorophyll traits of two wheat genotypes that differ in PUE. RAC875 was a P efficient genotype and Wyalkatchem was a P inefficient genotype. The plants were grown in pots under growth room conditions at two P levels; 10 mg P kg^–1^ soil (low P) and 30 mg P kg^–1^ soil (adequate P) and gas exchange and chlorophyll fluorescence were measured at the vegetative and booting stages using a portable photosynthesis system (LI-6800, LI-COR, United States). Results showed significant differences in some agronomic traits between the two wheat genotypes, i.e., greater leaf size and area, and a higher ratio of productive tillers to total tillers in RC875 when compared with Wyalkatchem. The CO_2_ response curve showed Wyalkatchem was more severely affected by low P than RAC875 at the booting stage. The relative ratio of the photosynthetic rate at low P to adequate P was also higher in RAC875 at the booting stage. Photochemical quenching (qP) in RAC875 was significantly higher when compared with Wyalkatchem at the booting stage. Maintaining CO_2_ fixation capacity under low P and higher qP would be associated with P efficiency in RAC875 and measuring qP could be a potential method to screen for P efficient wheat.

## Introduction

Phosphorus (P) is an essential macronutrient for higher plants and an important fertilizer for crops, but P fertilizer sources are being depleted (Vance et al., 2003). Upon application to soil, P fertilizer is rapidly immobilized due to chemical fixation and microbial immobilization (Indiati and Sharpley, 1998). It has been found that less than 20% of P fertilizer is available to be freely taken up by crops (Indiati and Sharpley, 1998).

Phosphorus deficiency significantly reduces crop grain yield and biomass (Ozturk et al., 2005; McDonald et al., 2015). This nutrient is an essential component of ATP, nicotinamide adenine dinucleotide phosphate (NADPH), nucleic acids, and phospholipids in cell membranes, which are important components of photosynthesis and therefore P deficiency affects plant photosynthesis and then plant yield (Carstensen et al., 2018).

Photosynthesis is the process in which plants absorb light energy and this is transformed into chemical energy *via* carbon dioxide (CO_2_) fixation. The absorbed light has three potential fates: (1) it is used to drive photosynthesis, (2) released as heat, and (3) reemitted as fluorescence (Maxwell and Johnson, 2000). These are competition processes and how they compete with one another will affect the crop yield and biomass. Chlorophyll fluorescence measurements can be used to measure photochemistry (photosynthesis) and non-photochemistry (dissipated heat). The quantum efficiency of photosystem II (PSII; Φ_PSII_), photochemical quenching (qP), and electron transport rate (ETR) are indicators for the efficiency of photosynthesis, while non-photochemical quenching (NPQ) is an indicator of dissipated heat (Murchie and Lawson, 2013). Gas exchange such as photosynthetic rate can also be used to evaluate the photosynthetic potential of plants (Johnson and Murchie, 2011). For example, a recent study in wheat showed a positive correlation between grain yield and photosynthetic rate at the heading and grain filling stage (Yu et al., 2020). A similar result in wheat grown under well-watered conditions was also reported by Wasaya et al. (2021).

Revealing how plant photosynthesis responds to P deficiency is the first step to explore mechanisms of plant adaptation to low P supply through the adjustment of photosynthesis. Studies in rice and sheepgrass [*Leymus chinensis* (Trin.) Tzvel] showed that low P supply reduced photosynthetic rate (P_n_), maximum quantum efficiency of PSII (F_v_/F_m_), Φ_PSII_, and ETR, and in parallel, low P activates the NPQ mechanism to release excess light energy and results in increased NPQ (Xu et al., 2007; Li et al., 2018). A similar result was found in soybean (Chu et al., 2018). These results come from measurements taken at an early growth stage, while it has also been confirmed in older plants (50 days after seedling emergence) indicating that low P reduced P_n_ (Veronica et al., 2017). Also, only more extreme levels of P deficiency reduced Φ_PSII_ and ETR and increased NPQ (Veronica et al., 2017).

A number of studies have investigated how plants adapt to low P soils and improving our understanding of the mechanisms involved in phosphorus use efficiency (PUE) (Ganie et al., 2015; Deng et al., 2018; Nguyen et al., 2019; Nguyen and Stangoulis, 2019). These studies aim to assist in generating target traits for the development of P efficient crops; traits such as P acquisition efficiency (PAE, amount of P uptake per plant) or P utilization efficiency (PUtE, yield produced per unit of absorbed P), or the combination of these two characteristics. Improving our understanding of genotypic variation in response to P deficiency impacts on photosynthesis can help to identify useful traits for plant breeding purposes. A study in soybean found associations between PUE and gas exchange parameters (photosynthetic rate, stomatal conductance, transpiration rate, and intercellular CO_2_ concentration) (Li et al., 2016). However, information on the linkage between PUE and chlorophyll fluorescence is lacking and requires further research to improve our knowledge of mechanisms.

As reported in previous studies, RAC875 is a P efficient wheat genotype, while Wyalkatchem is a P inefficient wheat genotype (Nguyen et al., 2019; Nguyen and Stangoulis, 2019). Also, in a previous study, the P efficient genotype had a greater ability to maintain phosphorylated sugars (i.e., glucose-6-P and fructose-6-P) (Nguyen et al., 2019) that are important for glycolysis as well as the biosynthesis of sugars and starch (Bahaji et al., 2014; Li et al., 2015). This can result from efficiencies in the photosynthetic apparatus within the P efficient genotype. This study aimed to explore further the efficiency within the photosynthetic apparatus of the two wheat genotypes, RAC875 and Wyalkatchem. It also investigates whether RAC875 harbors favorable gas exchange and chlorophyll fluorescence characteristics that may be further exploited as indicators for selection in breeding for PUE in wheat.

## Materials and Methods

### Experimental Design

Two wheat genotypes RAC875 (P efficient) and Wyalkatchem (P inefficient) were used for this experiment. Plants were grown in round pots (18.5 cm deep × 17.5 cm top diameter × 16.0 cm base diameter) filled with 4.2 kg of a washed sand (washed concrete sand, commercially available). The plants were grown at two P rates, 10 and 30 mg P kg^–1^ soil (in the form of KH_2_PO_4_) along with added basal nutrients with the final amount in the soil as described in Table 1. Individual nutrient stocks were made and then the two nutrient solutions [designated solution 1 and solution 2 (Table 1) for 10 and 30 mg P kg^–1^ soil, respectively] were prepared. A quantity of 420 mL of the nutrient solution was added to preweighed, bagged, dry soil (4.2 kg) and mixed thoroughly by hand.

**TABLE 1:** Composition of two nutrient solutions and the final amount in soil when added as 100 ml aliquot.

Nutrient	Nutrient concentrations in solution 1 (mg 100 mL^–1^)	Nutrient concentrations in solution 2 (mg 100 mL^–1^)	Final amount in soil (mg kg^–1^)
*Ca(NO_3_)_2_	918	918	918
K_2_SO_4_	113.6	113.6	113.6
MgSO_4_⋅7H_2_O	140	140	140
FeSO_4_⋅7H_2_O	1.4	1.4	1.4
Na_2_MoO_4_⋅2H_2_O	0.61	0.61	0.61
CuSO_4_⋅5H_2_O	2.25	2.25	2.25
MnSO_4_⋅4H_2_O	3.68	3.68	3.68
ZnSO_4_⋅7H_2_O	6.6	6.6	6.6
H_3_BO_3_	0.28	0.28	0.28
P (in KH_2_PO_4_)	10.0	30.0	10.0 or 30.0

*Ca(NO_3_)_2_ was the last nutrient to be added into the solution (after all other nutrients and water were added) to avoid the precipitation of CaSO_4_ at high concentration.

Wheat seeds were washed thoroughly with Milli-Q^®^ (>18 MΩ resistivity) water, placed in Petri dishes lined with moistened filter paper, sealed with parafilm, and kept in a dark place at room temperature for 3 days. Two germinated seeds were sown into each pot, with four replicates of each treatment. The pots were kept in a controlled environment growth room with the following conditions: 20°C/10°C, 13-h/11-h day/night cycle, and light intensity of 700 μmol m^–2^ s^–1^ at the leaf level. Light source was a combination of fluorescent and incandescent lights. The pots were arranged in a randomized design and were rotated every 3–7 days to minimize the effect of light gradients within the growth room. One plant was thinned from each pot at 10 days after sowing (DAS). The plants were watered to 10% of the dry soil weight every 2–3 days.

### Gas Exchange Measurements

Gas exchange was measured using a portable photosynthesis system (LI-6800, LI-COR, United States) at the vegetative and booting stage (Supplementary Figure 1). The measurements were taken from 10:00 to 12:00 h (the day light hours were from 6 a.m. to 7 p.m.). At the vegetative stage, gas exchange was measured at 34 and 41 DAS and the measurements were made at the middle of the youngest fully expanded leaves. At the booting stage, the measurements were taken on the flag leaves. The two wheat genotypes varied in developmental stage in which RAC875 and Wyalkatchem reached booting stage at 55 and 69 DAS, respectively. The light source was the Multiphase Flash™ Fluorometer (6800-01A, LI-COR, United States) and light intensity was set at 700 μmol m^–2^s^–1^ (90% red and 10% blue) with an aperture of 6 cm^2^. All photosynthetic measurements were taken at a constant airflow rate of 500 μmol s^–1^. The CO_2_ concentration supplied was 400 μmol mol^–1^ and the temperature (Tleaf) was 22°C. The chamber humidity was controlled by setting the leaf vapor pressure deficit (VDP_leaf_) at 1. The measured leaf area was adjusted by the method developed by Savvides and Fotopoulos (2018).

Dark respiration was measured on dark-adapted leaves after 60 min wrapped with aluminum (Al) foil. The same setting parameters were used for the chamber as detailed above with no light illumination.

### Establishing the Carbon Dioxide Response Curve

The CO_2_ response curves were generated at the booting stage (55 DAS for RAC875 and 69 DAS for Wyalkatchem) on two replicates of each treatment for both genotypes. The light intensity was set at 1,500 μmol m^–2^s^–1^ (90% red and 10% blue) and the airflow rate was set at 500 μmol s^–1^. The temperature at the leaf surface (Tleaf) was set at 22°C. The CO_2_ concentrations were set at 400, 300, 200, 100, 50, 0, 400, 400, 600, 800, 1,000, and 1,200 μmol mol^–1^ using the default method of the instrument. The measurements were taken using the Auto Program and the logging time was set from 1 to 2 min.

### Chlorophyll Fluorescence Measurements

**FIGURE 1:**
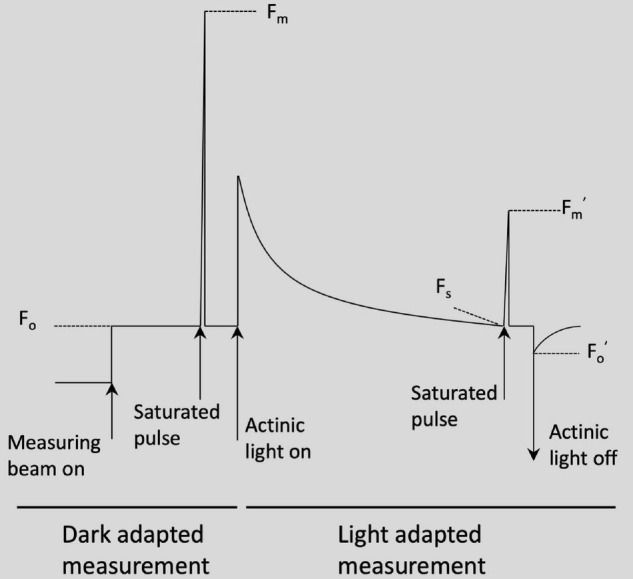
Chlorophyll fluorescence trace of dark adapted measurement and light adapted measurement [adapted from Maxwell and Johnson (2000)].

Chlorophyll fluorescence parameters were measured using a portable photosynthesis system (LI-6800, LI-COR, United States) and were taken on both dark-adapted and light-adapted leaves. For the dark-adapted measurement, the leaves were adapted to dark by wrapping with Al foils for 1 h before the measurement. The measurement was taken with the actinic light off and dark mode rate set to 50 Hz. The flash was rectangular with the red target 8,000 μmol m^–2^s^–1^, duration 1,000 ms, output rate of 100 Hz, and margin of 5 points. In this process, minimal fluorescence (F_o_) was measured before flash and maximal fluorescence (F_m_) was measured when flashed (Figure 1). The variable fluorescence and maximum quantum yield of PSII were calculated as shown in Table 2. For the light-adapted measurement, the leaves were adapted to light at least 1 h before the measurement. The actinic light was on and set at 534 μmol m^–2^s^–1^ (90% red and 10% blue) and dark mode rate, light mode rate, and flash mode rate were set to 50 Hz, 50 kHz, and 250 kHz, respectively. The flash was rectangular with the red target 8,000 μmol m^–2^s^–1^, duration 1,000 ms, output rate 100 Hz, and margin 5 points. The dark pulse parameters were set at 25 μmol m^–2^s^–1^ for far red target, 5 s for time that actinic light is off, 1 s for time that far red is off after actinic is off, 1 s for time that far red off after actinic is off, and 5 for number of data points before and after. The maximal fluorescence (F_m_’) was taken at the saturated flash period, then the steady state fluorescence (F_s_) was measured immediately before the flash, and minimal fluorescence (F’_o_) was measured after the flash and removal of actinic light (Figure 1). Photochemical efficiency of PS II (ΦPSII), qP, the ETR, and NPQ were calculated as described in Table 2; Maxwell and Johnson (2000).

**TABLE 2:** List of chlorophyll fluorescence parameters and equations [adapted from Maxwell and Johnson (2000)].

Parameter	Equation	Description
F_v_	F_v_ = F_m_-F_o_	Variable fluorescence
F_v_/F_m_	F_v_/F_m_ = (F_m_-F_o_)/F_m_	Maximum efficiency of PSII
Φ_PSII_	Φ_PSII_ = (F_m_’-F_s_)/F_m_’	Quantum yield of PSII
qP	qP = (F_m_’-F_s_)/(F_m_’-F_o_’)	Photochemical quenching, relates to maximum efficiency of PSII and relates to proportion of PSII centers that are open
ETR(*)	PFDa*Φ_PSII_*0.5	Electron transport rate
NPQ	NPQ = (F_m_-F_m_’)/F_m_’	Non-photochemical quenching, estimates the rate of heat loss from PSII

*PFDa is absorbed light and 0.5 is a correction factor that account for the partitioning of energy.

The airflow rate was set at 500 μmol s^–1^, CO_2_ concentration was set at 400 μmol mol^–1^, and the leaf temperature at 22°C. The humidity of chamber was controlled by setting VDP_leaf_ = 1. Measurements were made at the vegetative stage (42 DAS for both genotypes) and booting stage (53 for RAC875 and 68 DAS for Wyalkatchem).

### Leaf Area Measurement

Leaf area (LA) was measured using the method described by Wang et al. (2019). Maximal leaf width and leaf length were measured using a ruler with the accuracy of 1 mm. The LA was measured on the two youngest fully expanded leaves. The LA was calculated by the following equation (Wang et al., 2019).


$$\text{LA}=\frac{Leafwidth^{*}Leaflength}{1.2}$$

### Statistical Analysis

Statistical analysis was performed using R (version 4.0.3). Two-way ANOVA analysis was used to analyze the effect of genotype (G) and P supply (P) on agronomic traits (productive tiller number, productive tiller number/total tiller number, leaf number per main tiller, and grain yield), gas exchange parameters at the booting stage, and chlorophyll fluorescence parameters at the vegetative and booting stage were performed by a two-way ANOVA. Main effect differences were analyzed using a list significant different (LSD) test with Bonferroni correction (*P* < 0.05). Simple effect and interaction analysis were then performed to compare differences between genotypes at each P supply for these traits using the Phia package developed by De Rosario Martinez (2015). The effect of genotype, P supply and growth stage on tiller number, and gas exchange traits at the vegetative stage were analyzed using three-way ANOVAs. Main effect differences were analyzed using the LSD test with Bonferroni correction (*P* < 0.05). Simple interactions and simple effects were performed to analyze the interactions and effects for two variables at a specific level of the third variable (Kirk, 2014; UCLA, n.d.). Simple effects were also performed to analyze the effects for one variable at a specific level of the other two variables. The error term for simple interaction and simple effect analysis was corrected with the error term of entire data. The *F* statistic for a significant effect was corrected using two methods, the family error rate and Dun’s method; both values are reported to see if the statistical significance was consistent between the two methods (Kirk, 2014).

## Results

### Effect of Phosphorus Supply on Agronomic Traits in RAC875 and Wyalkatchem

**FIGURE 2:**
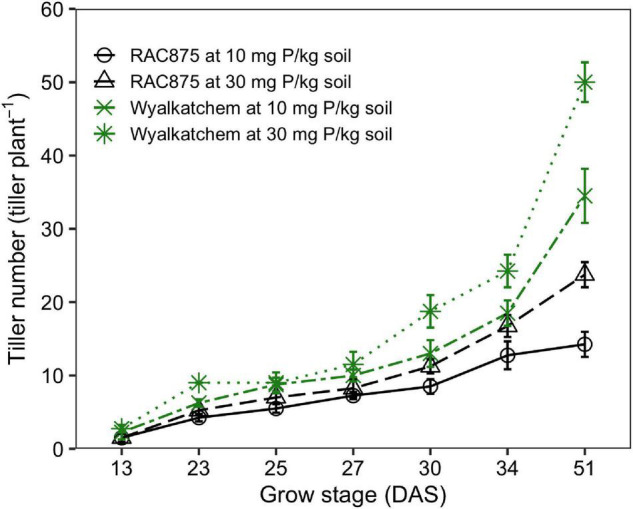
Effect of phosphorus (P) supply on the tiller number of two wheat genotypes, RAC875 and Wyalkatchem at various growth stages. Results are the means ± SD (*n* = 4).

Tiller number was significantly (*P* < 0.001) affected by P supply and tiller number also significantly (*P* < 0.001) varied according to genotype and growth stage (Figure 2 and Supplementary Table 1). Tiller development was slow from 13 to 27 DAS and then production dramatically increased until 51 DAS as can be seen in Figure 2.

Significant G × P × GS (*P* < 0.01), G × P (*P* < 0.05), G × GS (*P* < 0.001), and P × GS (*P* < 0.001) interactions for the tiller number were observed (Supplementary Table 1). Separate two-way ANOVAs found significant (*P* < 0.05 with the family error rate and Dunn’s method corrections) G × P interaction occurring at 69 DAS, but not at all the other growth stages. Tiller production responding to P was observed from 23 DAS where both wheat genotypes had more tiller numbers at adequate P when compared to low P (*P* < 0.05). The response was slightly less at 25 and 27 DAS, and then more obvious at older stages. Indeed, at 51 DAS, tiller numbers produced by plants grown under adequate P was 37 ± 14 on average, about 1.50-fold higher than under low P, while at 23 DAS, the number of tillers under adequate P was 10 ± 2, just about 1.14-fold higher than under low P (Figure 2).

**FIGURE 3:**
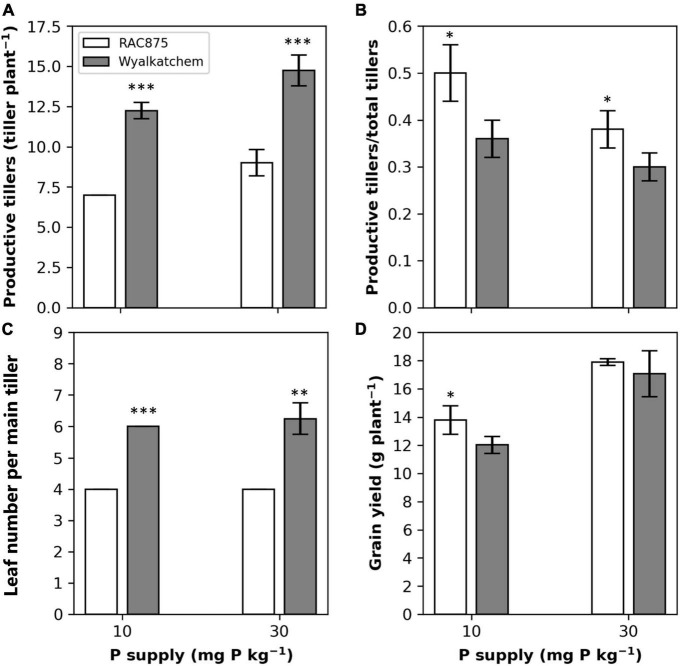
Effect of P supply on productive tillers **(A)**, the ratio of productive tillers to total tiller number **(B)**, leaf number per main tiller **(C)** at 69 days after sowing (DAS) [for all **(A–C)**], and grain yield **(D)** of two wheat genotypes, RAC875 and Wyalkatchem. Results are the means ± SD (*n* = 4). *, **, *** significant within the same P supply at *P* < 0.05, *P* < 0.01, *P* < 0.001 respectively.

No significant genotypic difference in the tiller number was observed at 13 DAS, but Wyalkatchem had significantly (*P* < 0.05 with the family error rate and Dunn’s correction) greater tiller number than RAC875 at all later growth stages (Figure 2 and Supplementary Table 1). At 51 DAS, the tiller number of RAC875 was 14 ± 2 and 24 ± 2 at low and adequate P, respectively, while the numbers were more than double for Wyalkatchem, 35 ± 4 and 50 ± 3 at low and adequate P, respectively. There were significant differences in productive tillers between genotypes; Wyalkatchem had 12 ± 1 (*P* < 0.001) and 13 ± 1 (*P* < 0.001) productive tillers greater than RAC875 that had 7 ± 0 and 9 ± 1 under low P and adequate P, respectively (Figure 3A).

Although Wyalkatchem had greater total tiller number as well as more productive tiller number than RAC875, RAC875 showed a significantly (*P* < 0.001) greater ratio of productive tillers to total tillers (Figure 3B). These ratios in RAC875 were 0.50 ± 0.06 and 0.38 ± 0.04, which were about 1.4-fold and 1.3-fold greater than those in Wyalkatchem at low and adequate P, respectively.

Leaf width, leaf length, and leaf area responded to P supply at 27 DAS where low P showed a significant (*P* < 0.001) reduction in leaf width and leaf area but not in leaf length for the youngest leaf, low P did not affect leaf width, leaf length, and leaf area for the second youngest leaf (Table 3). At 27 DAS, RAC875 had significantly (*P* < 0.001) greater leaf width, leaf length, and leaf area for the youngest and the second youngest leaf, when compared to Wyalkatchem (Table 3). However, RAC875 produced less leaf numbers per tiller than Wyalkatchem. At 69 DAS, both genotypes had flag leaves and RAC875 had 4 leaves per tiller, but Wyalkatchem had six leaves per tiller (Figure 3C).

**TABLE 3:** The effect of phosphorus (P) supply on leaf width, leaf length, and leaf area of the youngest and the second youngest leaf of two wheat genotypes, RAC875 and Wyalkatchem, at 27 days after sowing (DAS).

P treatment (mg P kg^–1^ soil)		The youngest leaf			The second youngest leaf		
		Leaf width (cm)	Leaf length (cm)	Leaf area (cm^–2^)	Leaf width (cm)	Leaf length (cm)	Leaf area (cm^–2^)
10	RAC875	1.05 ± 0.04^a^	27.0 ± 0.99^a^	23.7 ± 0.99^a^	0.95 ± 0.09^a^	26.5 ± 2.63^a^	24.2 ± 4.30^a^
	Wyalkatchem	0.91 ± 0.06^b^	24.6 ± 0.62^b^	18.6 ± 1.63^b^	0.76 ± 0.10^b^	21.0 ± 0.91^b^	13.4 ± 2.22^b^
30	RAC875	1.25 ± 0.05^a^	29.9 ± 1.03^a^	31.1 ± 1.70^a^	1.09 ± 0.03	26.4 ± 0.97^a^	23.9 ± 1.21^a^
	Wyalkatchem	0.99 ± 0.09^b^	23.2 ± 1.54^b^	19.0 ± 0.56^b^	0.94 ± 0.10	22.4 ± 2.56^b^	17.5 ± 3.04^b^
*P-*value							
Genotype (G)		*P* < 0.001	*P* < 0.001	*P* < 0.001	*P* < 0.001	*P* < 0.001	*P* < 0.001
P supply (P)		*P* < 0.001	*P* = 0.1875	*P* < 0.001	*P* = 0.0695	*P* = 0.5102	*P* = 0.202
G × P		*P* = 0.0927	*P* = 0.0015	*P* < 0.001	*P* = 0.0695	*P* = 0.4498	*P* = 0.156

*Results are the means ± SD (n = 4). Different letters show significant differences between genotypes within the same P supply (P < 0.05).*

Low P significantly (*P* < 0.001) reduced grain yield and the reduction occurred more so in Wyalkatchem (Figure 3). RAC875 showed 14.6% greater (*P* < 0.05) grain yield than Wyalkatchem under low P, while no significant difference in grain yield between the two genotypes was observed under adequate P (Figure 3D and Supplementary Table 2).

### Effect of Phosphorus Supply and Growth Stage on Gas Exchange Traits in RAC875 and Wyalkatchem

**FIGURE 4:**
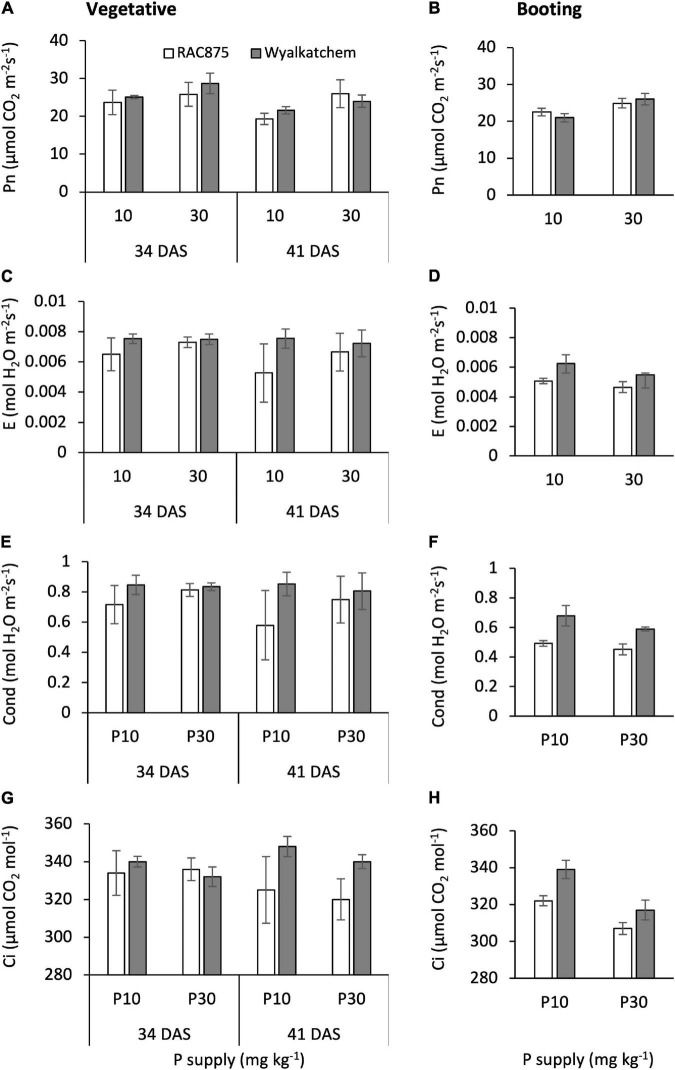
The effects of P supply on photosynthetic rate (Pn) at the vegetative **(A)** and booting **(B)** stage, transpiration rate (E) at the vegetative **(C)** and booting **(D)** stage, stomatal conductance (Cond) at the vegetative **(E)** and booting **(F)** stage, and intercellular CO_2_ concentration (Ci) at the vegetative **(G)** and booting **(H)** stage of RAC875 and Wyalkatchem. The results are the means ± SD (*n* = 4).

Three-way ANOVA analysis showed that at the vegetative stage, genotype had no significant effect on photosynthetic rate, but it significantly (*P* < 0.05) impacted transpiration rate, stomatal conductance, and intercellular CO_2_ concentration. In contrast, P supply significantly (*P* < 0.05) affected photosynthetic rate, while it had no significant effect on transpiration rate, stomatal conductance, and intercellular CO_2_ concentration (Table 4). Growth stage at the vegetative stage also showed a significant (*P* < 0.05) effect on photosynthetic rate, but not on the other three gas exchange parameters (Table 4). No significant G × P × GS, G × P, and P × GS interactions were found for the four gas exchange parameters. Significant (*P* < 0.05) G × GS interaction for intercellular CO_2_ concentration was observed (Table 4, Figure 4G), while no significant interactions were found for photosynthetic rate, transpiration rate, and stomatal conductance.

**TABLE 4:** Analysis of variance for the effects of genotype, P supply, and growth stage on photosynthetic rate (P_n_), transpiration rate (E), stomatal conductance (Cond), intercellular CO_2_ concentration (C_i_), and at the vegetative stage.

Genotype	P_n_ (μmol CO_2_ m^–2^s^–1^)	E (mol H_2_O m^–2^s^–1^)	Cond (mol H_2_O m^–2^s^–1^)	C_i_ (μmol CO_2_ m^–2^s^–1^)
*P-*value				
Genotype (G)	*P* = 0.187	*P* = 0.008	*P* = 0.010	*P* = 0.014
P supply (P)	*P* < 0.001	*P* = 0.213	*P* = 0.238	*P* = 0.480
Growth stage (GS)	P = 0.001	*P* = 0.147	*P* = 0.207	*P* = 0.982
G × P	*P* = 0.420	*P* = 0.084	*P* = 0.072	*P* = 0.092
G × GS	*P* = 0.250	*P* = 0.259	*P* = 0.313	*P* = 0.028
P × GS	*P* = 0.324	*P* = 0.838	*P* = 0.824	*P* = 0.836
G × P × GS	*P* = 0.104	*P* = 0.544	*P* = 0.535	*P* = 0.870

On an average, at the vegetative stage, Wyalkatchem had significantly (*P* < 0.05) higher transpiration rate and stomatal conductance than RAC875. Transpiration rate and stomatal conductance in Wyalkatchem were 0.0075 ± 0.0005 and 0.83 ± 0.07 mol H_2_O m^–2^s^–1^, respectively, while these values in RAC875 were 0.0064 ± 0.0010 and 0.71 ± 0.16 mol H_2_O m^–2^s^–1^ (Figures 4C,E and Table 4).

At the vegetative stage, on an average, low P significantly (*P* < 0.05) reduced the photosynthetic rate to 22.4 ± 2.8 μmol CO_2_ m^–2^s^–1^ in comparison with 26.1 ± 3.1 μmol CO_2_ m^–2^s^–1^ at adequate P. At this stage, on an average, photosynthetic rate at 34 DAS (25.8 ± 3.0 μmol CO_2_ m^–2^s^–1^) was significantly (*P* < 0.05) higher than that at 41 DAS (22.7 ± 3.3 μmol CO_2_ m^–2^s^–1^) (Figure 4A and Table 4).

A significant (*P* < 0.05) P × GS interaction occurred for intercellular CO_2_ concentration, therefore further separate two-way ANOVAs at each growth stage were used and the result showed genotype significantly affected intercellular CO_2_ concentration at 41 DAS (*P* < 0.05 with both family error rate and Dunn’s corrections), while genotype did not impact this parameter at 34 DAS. Wyalkatchem had greater intercellular CO_2_ concentration at 41 DAS than RAC875 (Supplementary Table 3).

At the booting stage, genotype showed no significant effect on photosynthetic rate, while genotype significantly (*P* ≤ 0.001) affected transpiration rate, stomatal conductance, and intercellular CO_2_ concentration (Table 5). At this growth stage, P supply had a significant (*P* < 0.01) effect on the four gas exchange parameters. No significant G × P interactions occurred for transpiration rate, stomatal conductance, and intercellular CO_2_ concentration, while a slight (*P* = 0.062) G × P interaction for photosynthetic rate was observed. This explained the greater reduction in photosynthetic rate under low P in Wyalkatchem compared with RAC875 at the booting stage (Figure 4B); the relative ratio of photosynthetic rate at low P to adequate P in Wyalkatchem (0.83) was greater than in RAC875 (0.90) (Supplementary Table 4).

**TABLE 5:** ANOVA for the effects of genotype and P supply on photosynthetic rate (P_n_), transpiration rate (E), stomatal conductance (Cond), intercellular CO_2_ concentration (C_i_), and at the booting stage.

Genotype	P_n_ (μmol CO_2_ m^–2^s^–1^)	E (mol H_2_O m^–2^s^–1^)	Cond (mol H_2_O m^–2^s^–1^)	C_i_ (μmol CO_2_ m^–2^s^–1^)
*P-*value				
Genotype (G)	*P* = 0.761	*P* = 0.001	*P* < 0.001	*P* < 0.001
P supply (P)	*P* < 0.001	*P* = 0.007	*P* = 0.008	*P* < 0.001
G × P	*P* = 0.062	*P* = 0.372	*P* = 0.255	*P* = 0.125

At the booting stage, Wyalkatchem had a significantly (*P* < 0.05) higher transpiration rate, stomatal conductance, and intercellular CO_2_ concentration than RAC875 (Figures 4D,F,H). Transpiration rate, stomatal conductance, and intercellular CO_2_ concentration were 0.0059 ± 0.0006 mol H_2_O m^–2^s^–1^, 0.64 ± 0.07 mol H_2_O m^–2^s^–1^, and 328 ± 13 μmol CO_2_ mol^–1^, respectively, while these values in RAC875 were 0.0049 ± 0.0004 mol H_2_O m^–2^s^–1^, 0.47 ± 0.03 mol H_2_O m^–2^s^–1^, and 314 ± 9 μmol CO_2_ mol^–1^, respectively.

**FIGURE 5:**
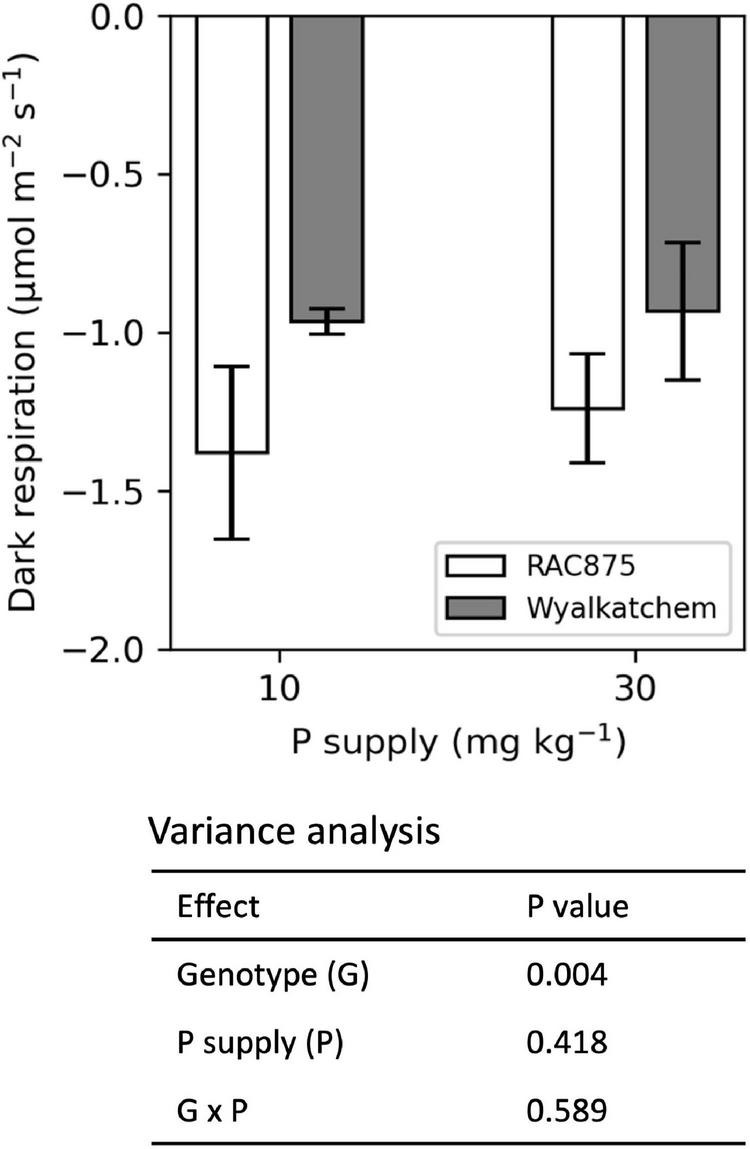
Effect of genotype and P supply on dark respiration at booting stage. The results are the means ± SD (*n* = 4).

Dark respiration was measured at the booting state and the results showed that P supply did not significantly affect dark respiration, while significant (*P* < 0.01) variation in the dark respiration between genotypes was observed (Figure 5). The G × P interaction was not significant for dark respiration. A LSD test showed RAC875 had significantly (*P* < 0.05) greater dark respiration (−1.30 ± 0.21 μmol CO_2_ m^–2^s^–1^) than Wyalkatchem (−0.95 ± 0.15 μmol CO_2_ m^–2^s^–1^).

**FIGURE 6:**
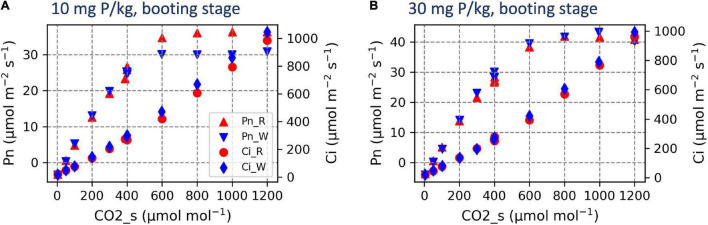
CO_2_ response curves of two wheat genotypes under different P treatments at the booting stage under low P **(A)** and adequate P **(B)**. The results are the mean of two replicates. Pn_R and Pn_W are the photosynthetic rate of RAC875 and Wyalkatchem, respectively; Ci_R and Ci_W are the CO_2_ intercellular concentration of RAC875 and Wyalkatchem, respectively.

The CO_2_ response curve displaying the relationship between intercellular CO_2_ concentration and photosynthetic rate was generated at the booting stage. The results showed that at adequate P, plants reached maximal photosynthetic rate at the chamber CO_2_ concentration of 800 and 1,000 μmol mol^–1^ for RAC875 and Wyalkatchem, respectively, corresponding to the intercellular CO_2_ concentration of about 600 and 800 μmol CO_2_ mol^–1^ (Figure 6B). In contrast, under low P, RAC875 reached maximum photosynthetic rate at the chamber CO_2_ concentration of 800 μmol mol^–1^, corresponding to the intercellular CO_2_ concentration of about 600 μmol CO_2_ mol^–1^, while Wyalkatchem reached maximum photosynthetic rate at the chamber CO_2_ concentration of 600 μmol mol^–1^, corresponding to the intercellular CO_2_ concentration of about 400 μmol CO_2_ mol^–1^ (Figure 6A). At the same chamber CO_2_ concentration, Wyalkatchem tends to have higher intercellular CO_2_ concentration.

### Effect of Phosphorus Supply and Growth Stage on Chlorophyll Fluorescence Traits in RAC875 and Wyalkatchem

Six chlorophyll fluorescence parameters including F_o_, F_m_, F_v_, F_v_/F_m_, Φ_PSII_, and ETR were measured at the vegetative and booting stage and two parameters, NPQ and qP, were measured at the booting stage. The results showed that at both the vegetative and booting stage, the genotype significantly (*P* < 0.05) affected F_o_, F_m_, F_v_, and F_v_/F_m_, while it had a slight (*P* = 0.066) impact on Φ_PSII_ and ETR at the vegetative stage and had no significant effect on these two parameters at the booting stage (Tables 6A,B). At the booting stage, the genotype had no significant effect on NPQ, but it showed a significant (*P* < 0.05) effect on qP.

**TABLE 6:** ANOVA for the effects of genotype and P supply and growth stage on F_o_, F_m_, F_v_, F_v_/F_m_, Φ_PSII,_ ETR, NPQ, and qP at vegetative stage **(A)** and booting stage **(B)**.

(A) Vegetative stage						
Genotype	F_0_	F_m_	F_v_	F_v_/F_m_	Φ_PSII_	ETR
*P-*value						
Genotype (G)	*P* < 0.001	*P* < 0.001	*P* < 0.001	*P* = 0.028	*P* = 0.066	*P* = 0.066
P supply (P)	*P* = 0.004	*P* = 0.001	*P* < 0.001	*P* = 0.251	*P* = 0.714	*P* = 0.714
G × P	*P* = 0.347	*P* = 0.106	*P* = 0.083	*P* = 0.072	*P* = 0.716	*P* = 0.716
**(B) Booting stage**								
**Genotype**	**F_0_**	**F_m_**	**F_v_**	**F_v_/F_m_**	**Φ_PSII_**	**ETR**	**NPQ**	**qP**

*P-*value								
Genotype (G)	*P* < 0.001	*P* < 0.001	*P* < 0.001	*P* = 0.030	*P* = 0.834	*P* = 0.834	*P* = 0.287	*P* = 0.019
P supply (P)	*P* = 0.246	*P* = 0.058	*P* < 0.042	*P* = 0.158	*P* = 0.182	*P* = 0.183	*P* = 0.131	*P* = 0.645
G × P	*P* = 0.010	*P* = 0.003	*P* = 0.003	*P* = 0.942	*P* = 0.257	*P* = 0.257	*P* = 0.094	*P* = 0.666

*The explanation for these parameters were described in Table 1.*

At the vegetative stage, P supply significantly influenced F_o_, F_m_, and F_v_, while it had no impact on F_v_/F_m_, Φ_PSII_, and ETR. At this stage, no significant P × G interactions were found for these six chlorophyll fluorescence parameters (Table 6A). However, at the booting stage, P supply had a significant effect on only F_v_, but not on the other five parameters (Table 6B). At the booting stage, significant G × P interaction for F_o_, F_m_, and F_v_ was observed, while no significant G × P interactions occurred for F_v_/F_m_, Φ_PSII_, and ETR.

**FIGURE 7:**
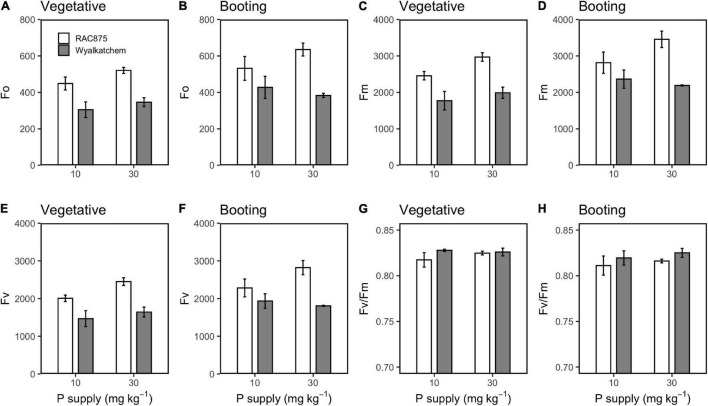
Effect of P supply on F_o_ at the vegetative **(A)** and booting **(B)** stage, F_m_ at the vegetative **(C)** and booting **(D)** stage, F_v_ at the vegetative **(E)** and booting **(F)** stage, and F_v_/F_m_ at the vegetative **(G)** and booting **(H)** stage in RAC875 and Wyalkatchem. Values represent the mean ± SD (*n* = 4).

**FIGURE 8:**
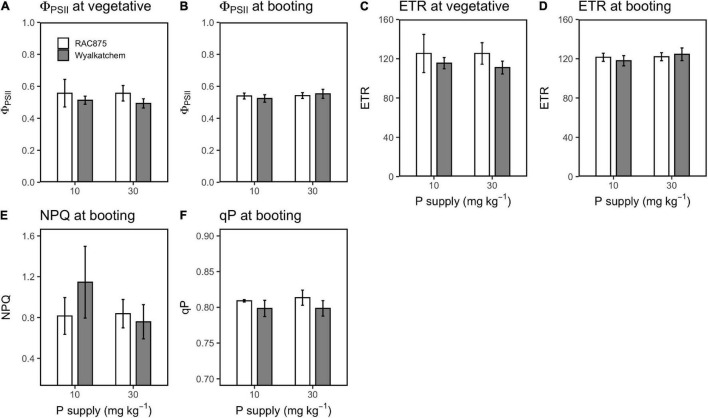
Effect of P supply on Φ_PSII_ at the vegetative **(A)** and booting **(B)** stage, ETR at the vegetative **(C)** and booting **(D)** stage, and NPQ **(E)** and qP **(F)** at the booting satge in RAC875 and Wyalkatchem. Values represent the mean ± SD (*n* = 4).

LSD analysis showed that RAC875 had significantly (*P* < 0.05) greater F_o_, F_m_, and F_v_ than Wyalkatchem at both vegetative and booting stages (Figures 7A–F), while Wyalkatchem showed significantly (*P* < 0.05) greater F_v_/F_m_ than RAC875 (Figures 7G,H). In contrast to F_v_/F_m_, qP in RAC875 was significantly (*P* < 0.05) higher than that in Wyalkatchem at booting stage (Figure 8F).

At the vegetative stage, low P significantly (*P* < 0.01) reduced F_o_, F_m_, and F_v_ (Figures 7A,C,E). These values at low P, on an average, were 377 ± 85, 2114 ± 409, and 1737 ± 325, respectively, while at adequate P, these values were 433 ± 95, 2480 ± 540, and 2047 ± 445, respectively. At the booting stage, significant G × P interactions occurred for F_o_, F_m_, and F_v_; therefore, further interaction analysis showed that low P significantly (*P* < 0.05) reduced F_o_, F_m_, and F_v_ for RAC875 but not for Wyalkatchem (Figures 7B,D,F and Supplementary Table 5).

Genotype and P supply had no significant effect on NPQ. However, a slight (*P* = 0.094) P × G interaction occurred for this parameter. Further interaction analysis showed low increased (*P* = 0.063) NPQ 1.5-fold in Wyalkatchem, while low P did not increase NPQ in RAC875 (Supplementary Tables 6, 7).

## Discussion

Phosphorus is an important macronutrient for plants, but low P availability occurs in many agricultural lands and P fertilizer sources are being depleted. Therefore, identifying favorable traits for PUE in plants could make a contribution to solve this problem. This study found that a P efficient wheat genotype (RAC875) had agronomic traits that may explain its ability to succeed on P deficient soils such as a larger leaf area, lower relative leaf number per tiller, and a higher ratio of productive tillers to total tillers. RAC875 also had some differences in gas exchange and chlorophyll fluorescence traits that might help to contribute to its PUE. This discussion will explore how wheat genotypes respond to P levels and how contrasting agronomic, gas exchange and chlorophyll traits between the two wheat genotypes may help to explain PUE in RAC875.

### Differences in Agronomic Traits Between the Phosphorus Efficient and the Phosphorus Inefficient Wheat

#### Elevated Tiller Production at Vegetative Stage and High Tiller Mortality Could Result in Lower Grain Yield Under Low Phosphorus in Wyalkatchem

Wyalkatchem produced a large number of tillers during the vegetative stage and many tillers were not productive. In contrast, RAC875 produced less tillers during the vegetative stage and it had a lower tiller mortality. Chen et al. (2019) also found that excessive tiller production at elongation stage results in higher unproductive tillers or higher mortality. Although this genotype had greater tiller number than RAC875, increased tiller production during the vegetative state could lead to a lower ratio of fertile tillers to total produced tillers. A high level of P supply would be required for higher tiller production if no mechanisms of efficiency were present, and we know from previous reports that Wyalkatchem is P inefficient (Nguyen et al., 2019; Nguyen and Stangoulis, 2019). It is likely that the greater tiller production in Wyalkatchem during the early stages of growth could result in the lack of nutrition for later grain yield production in Wyalkatchem. Therefore, a reduced straw biomass (and higher harvest index) may be a favorable trait in plant breeding (Supplementary Table 8).

#### Does a Larger Leaf Area and Lower Leaf Number Contribute to Greater Phosphorus Use Efficiency in RAC875?

It seems that RAC875 and Wyalkatchem have different developmental approaches. RAC875 produced a lower leaf number but with greater leaf area, while Wyalkatchem had higher leaf number with smaller leaf area (Table 3). Johnson et al. (1990) pointed out that high leaf number is not beneficial for yield production in wheat since leaf number is negatively correlated with grain yield. In contrast, flag leaf area is positively correlated with grain yield (Monyo and Whittington, 1973). Another study also found that flag leaf length, flag leaf width, and flag leaf area were positively correlated with yield-related traits in wheat (e.g., grain number per spike, grain weight per spike) (Ma et al., 2020). What are the reasons behind the positive correlation between area per leaf with grain yield and the negative correlation between leaf number and grain yield? We hypothesize that a large leaf area, particularly the flag leaf area, can capture more light for photosynthesis, and this helps to maintain grain yield. While more leaf numbers reduce grain yield since plants may allocate more nutrients to leaf production and therefore less nutrients are available to contribute to grain yield. This seems to fit with reasoning for P inefficiency in Wyalkatchem.

#### Differences in Gas Exchange and Chlorophyll Fluorescence Traits Between the Phosphorus Efficient and the Phosphorus Inefficient Wheat

Low P supply negatively impacts on photosynthesis and reduces its rate. This study showed a significant reduction in photosynthetic rate in both wheat genotypes under low P (Tables 4, 5 and Figures 4A,B). P is an important macronutrient and is involved in the synthesis of compounds associated with CO_2_ fixation (i.e., ATP). Indeed, low P reduces the efficiency of photosynthetic electron transport and results in a decreased ATP production (Lin et al., 2009). In turn, the decline of ATP decreases the regeneration of ribulose-1,5-bisphosphate (RuBP), which is vital for CO_2_ fixation (Rao and Terry, 1989; Rao et al., 1993). P deficiency also affects the activity of ribulose-1,5-bisphosphate carboxylase (RubisCO), which is a key enzyme for the dark phase of photosynthesis (Brooks, 1986; Brooks et al., 1988).

At the booting stage, the CO_2_ response curve showed that under low P, there was a more negative influence on Wyalkatchem when compared with RAC875 (Figure 6). Also, at the booting stage, the ratio of photosynthetic rate at low P to adequate P in RAC875 (0.90) was higher than in Wyalkatchem (0.83) (Figure 4B and Supplementary Table 4). In contrast, at the vegetative stage, the relative ratio of photosynthetic rate at low P to adequate P in RAC875 (0.83) was lower than in Wyalkatchem (0.89) (Supplementary Table 4). Maintaining the photosynthetic rate under low P compared to under adequate P at the later stage could contribute to greater grain yield in RAC875 under low P. This result is consistent with our previous finding where under low P, RAC875 was better in maintaining phosphorylated sugars necessary for the biosynthesis of sugars and starch (Nguyen et al., 2019). This result is similar to previous studies that showed that the grain yield was positively correlated with photosynthetic rate at heading and the grain filling stage in wheat (Yu et al., 2020), and at stages before flowering in sorghum (Peng et al., 1991).

Stomatal conductance is the rate of CO_2_ entering, or water vapor exiting through the stomatal pores of a leaf (Roche, 2015). Increased stomatal conductance is considered as a potential approach for higher grain yield in crops (Fischer et al., 1998; Bahar et al., 2009; Roche, 2015). However, this study showed that the greater grain yield of wheat in RAC875 under low P had a lower stomatal conductance than Wyalkatchem (Tables 4, 5 and Figure 4). Similarly, transpiration rate, an indicator of water loss, has a similar trend to stomatal conductance. A previous study found that RAC875 is a wheat genotype that consumes less water (Izanloo et al., 2008), and the results presented in this study are also able to show a lower stomatal conductance and transpiration rate in this genotype. The stomatal conductance and transpiration play different roles such as evaporative cooling, nutrient uptake from soil, CO_2_ entry, and water uptake (Sterling, 2005). The greater stomatal conductance and transpiration rate in Wyalkatchem may be associated with higher levels of water usage and nutrient requirements. In fact, Wyalkatchem is more responsive to water than RAC875 (Yadav et al., 2019) and had greater shoot P concentration than RAC875 (Nguyen, 2017).

The maximum photochemical efficiency (F_v_/F_m_) may reflect the maximum potential of plant photosynthesis (Xu et al., 2020) and genotypic differences in F_v_/F_m_ were identified in this study. Wyalkatchem had significantly higher F_v_/F_m_ than RAC875 (Tables 6A,B and Figures 7G,H). This indicates that Wyalkatchem is able to achieve greater photosynthetic capacity than RAC875, particularly under optimum conditions. The CO_2_ response curve at the booting stage showed that under adequate P, Wyalkatchem had a slightly higher photosynthetic rate compared with RAC875 when the CO_2_ concentration increased (Figure 6B). However, under low P, Wyalkatchem showed lower photosynthetic rate than RAC875 at high CO_2_ concentrations. This indicates that low P affected the photosynthetic system of Wyalkatchem more severely in comparison with RAC875 (Figure 6A). As a result, Wyalkatchem produced lower grain yield than RAC875 under low P.

During photosynthesis, the Φ_PS2_ measures the proportion of light absorbed by PSII reaction centers that is used in photochemistry, the ETR measures the rate of electron transport, and the qP measures the degree of openness of the reaction center during photosynthesis (Zhang et al., 2020). This study showed that low P had no significant effect on Φ_PS2_, ETR, and qP (Tables 6A,B and Figures 8A–D,F). These results are different from previous studies in rice in which these values reduced under P deficiency (Xu et al., 2007). This difference between two experiments could be due to differences of P treatment methods. Our experiment was a soil-based growth and plants were treated with two different P levels: low P (10 mg P kg^–1^ soil) and adequate P (30 mg P kg^–1^ soil), while the experiment conducted by Xu et al. (2007) was a hydroponic experiment and plants were treated with two different P levels: control (0 mM KH_2_PO_4_) and treatment (0.3 mM KH_2_PO_4_). No P present in the control could result in severe effects on the photosynthetic apparatus and thus lead to a significant reduction in Φ_PS2_, qP, and ETR. A study in peanut also showed that with more extreme P stress, the greater reduction in these parameters (Shi et al., 2020) was observed.

Although P deficiency had no significant effect on Φ_PS2_, ETR, and qP, RAC875 showed significantly higher (*P* < 0.05) qP at the booting stage and slightly higher (*P* = 0.066) Φ_PS2_, ETR at the vegetative stage than Wyalkatchem (Tables 6A,B and Figures 8A–D,F). Genotypic variations in these parameters were also observed in wheat (del Pozo et al., 2020) and in peanut (Shi et al., 2020). Φ_PS2_ and ETR have a similar trend since ETR is calculated from Φ_PS2_. Φ_PS2_ and ETR indicate overall photosynthesis, while qP refers to photochemical quenching in which the light energy is converted into chemical energy that is used later to drive photosynthesis (Ritchie, 2006). The relationship between these parameters and the crop grain yield as well as PUE in crops are limited. The greater qP in the P efficient wheat (RAC875) compared with the P inefficient wheat (Wyalkatchem) might be PUE indicators.

In contrast to qP, which indicates the process in which absorbed light energy is converted into chemical energy, NPQ is non-photochemical quenching, which indicates the process in which excess light energy is dissipated into heat (Ritchie, 2006; Ruban, 2016). NPQ is a protective mechanism to prevent the plant photosystem from damage under high light intensity. Although it is the protective approach of plants, heat dissipation leads to yield loss (Kromdijk et al., 2016). Studies found that low P increases NPQ in plants (Xu et al., 2007; Shi et al., 2020). However, this study showed that low P increased (*P* = 0.063) NPQ in Wyalkatchem but not in RAC875 (Table 6B and Figure 8E). It seems that Wyalkatchem is more sensitive to P deficiency than RAC875 and low P strongly affected the photosynthetic system of Wyalkatchem. This negative impact might cause more heat dissipation in Wyalkatchem and result in a significant reduction in grain yield under low P. Méndez-Espinoza et al. (2019) also reported that NPQ is negatively correlated with grain yield in wheat.

This study was conducted on two contrasting PUE wheat genotypes and revealed a favorable chlorophyll trait (qP) for PUE. Further research should be conducted on measuring qP of a large wheat population to classify P efficient wheat and P inefficient wheat based on this parameter. The CO_2_ response curve showed that low P had a more severe effect on the inefficient wheat, but low P showed slightly (*P* = 0.063) increased NPQ in the inefficient wheat. Thus, a study on a large wheat population would confirm if low P significantly increases NPQ under low P in inefficient genotypes. Also, elevated CO_2_ through climate change and P deficiency are current issues (Singh and Reddy, 2014; Manoj-Kumar et al., 2018) and the CO_2_ response curve indicates that low P had more of a negative effect on the photosynthetic rate of Wyalkatchem at elevated CO_2_. It would be interesting to study the responses of these two wheat genotypes to the combination effect of elevated CO_2_ and P deficiency in a larger, more controlled manner.

## Conclusion

This work has revealed that the P efficient wheat, RAC875, had favorable agronomic, gas exchange, and chlorophyll fluorescence traits in comparison with the P inefficient wheat, Wyalkatchem. RAC875 had greater leaf area per leaf and RAC875 was also more efficient in producing productive tillers than Wyalkatchem. Regarding gas exchange, low P showed more impact on Wyalkatchem than RAC875 at the booting stage. Regarding chlorophyll fluorescence, photochemical quenching (qP) was higher in RAC875 than in Wyalkatchem, while NPQ increased (*P* = 0.063) under low P in Wyalkatchem but not in RAC875. Low P was less affected on photosynthetic rate on RAC875 at the booting stage as well as the greater in qP might contribute to higher PUE in RAC875.

## Data Availability Statement

The original contributions presented in the study are included in the article/Supplementary Material, further inquiries can be directed to the corresponding author/s.

## Author Contributions

VN and JS designed the research. VN implemented the experiments, performed the data analysis, and wrote the manuscript. LP was involved in editing the manuscript and analyzing the data. JS made the revision of the manuscript. All the authors approved the final revision to be published.

## Conflict of Interest

The authors declare that the research was conducted in the absence of any commercial or financial relationships that could be construed as a potential conflict of interest.

## Publisher’s Note

All claims expressed in this article are solely those of the authors and do not necessarily represent those of their affiliated organizations, or those of the publisher, the editors and the reviewers. Any product that may be evaluated in this article, or claim that may be made by its manufacturer, is not guaranteed or endorsed by the publisher.

## References

[B1] Bahaji A. Li J. Sánchez-López ÁM. Baroja-Fernández E. Muñoz F. J. Ovecka M. (2014). Starch biosynthesis, its regulation and biotechnological approaches to improve crop yields. *Biotechnol. Adv.* 32 87–106. 10.1016/j.biotechadv.2013.06.006 23827783

[B2] Bahar B. Yildirim M. Barutcular C. (2009). Relationships between Stomatal Conductance and Yield Components in Spring Durum Wheat under Mediterranean Conditions. *Not. Bot. Horti Agrobot. Cluj Napoca* 37 45–48.

[B3] Brooks A. (1986). Effects of phosphorus nutrition on ribulose-1,5-bisphosphate carboxylase activation, photosynthetic quantum yield and amounts of some calvin-cycle metabolites in spinach leaves. *Aust. J. Plant Physiol.* 13 221–237.

[B4] Brooks A. Woo K. C. Wong S. C. (1988). Effects of phosphorus nutrition on the response of photosynthesis to CO_2_ and O_2_, activation of ribulose bisphosphate carboxylase and amounts of ribulose bisphosphate and 3-phosphoglycerate in spinach leaves. *Photosynth. Res.* 15 133–141. 10.1007/BF00035257 24430858

[B5] Carstensen A. Herdean A. Schmidt S. B. Sharma A. Spetea C. Pribil M. (2018). The Impacts of Phosphorus Deficiency on the Photosynthetic Electron Transport Chain. *Plant Physiol.* 177:271. 10.1104/pp.17.01624 29540590 PMC5933119

[B6] Chen X.-X. Zhang W. Liang X.-Y. Liu Y.-M. Xu S.-J. Zhao Q.-Y. (2019). Physiological and developmental traits associated with the grain yield of winter wheat as affected by phosphorus fertilizer management. *Scientific Rep.* 9:16580. 10.1038/s41598-019-53000-z 31719561 PMC6851383

[B7] Chu S. Li H. Zhang X. Yu K. Chao M. Han S. (2018). Physiological and Proteomics Analyses Reveal Low-Phosphorus Stress Affected the Regulation of Photosynthesis in Soybean. *Int. J. Mol. Sci.* 19:1688. 10.3390/ijms19061688 29882786 PMC6032344

[B53] De Rosario Martinez H. (2015). *Analysing Interactions of Fitted Models*. Available online at: https://cran.rproject.org/web/packages/phia/vignettes/phia.pdf

[B8] del Pozo A. Méndez-Espinoza A. M. Romero-Bravo S. Garriga M. Estrada F. Alcaíno M. (2020). Genotypic variations in leaf and whole-plant water use efficiencies are closely related in bread wheat genotypes under well-watered and water-limited conditions during grain filling. *Scientific Rep.* 10:460. 10.1038/s41598-019-57116-0 31949177 PMC6965644

[B9] Deng Y. Teng W. Tong Y.-P. Chen X.-P. Zou C.-Q. (2018). Phosphorus Efficiency Mechanisms of Two Wheat Cultivars as Affected by a Range of Phosphorus Levels in the Field. *Front. Plant Sci.* 9:1614. 10.3389/fpls.2018.01614 PMC6232341 30459796

[B10] Fischer R. A. Rees D. Sayre K. D. Lu Z. M. Condon A. G. Saavedra A. L. (1998). Wheat yield progress associated with higher stomatal conductance and photosynthetic rate, and cooler canopies. *Crop Sci.* 38 1467–1475.

[B11] Ganie A. H. Ahmad A. Pandey R. Aref I. M. Yousuf P. Y. Ahmad S. (2015). Metabolite Profiling of Low-P Tolerant and Low-P Sensitive Maize Genotypes under Phosphorus Starvation and Restoration Conditions. *PLoS One* 10:e0129520. 10.1371/journal.pone.0129520 PMC4474700 26090681

[B12] Indiati R. Sharpley A. N. (1998). Changes in distribution of inorganic soil phosphorus forms with phosphate desorption by iron oxide-impregnated paper strips. *Commun. Soil Sci. Plant Anal.* 29 625–634. 10.1080/00103629809369972

[B13] Izanloo A. Condon A. G. Langridge P. Tester M. Schnurbusch T. (2008). Different mechanisms of adaptation to cyclic water stress in two South Australian bread wheat cultivars. *J. Exp. Bot.* 59 3327–3346. 10.1093/jxb/ern199 18703496 PMC2529232

[B14] Johnson G. Murchie E. (2011). Gas exchange measurements for the determination of photosynthetic efficiency in Arabidopsis leaves. *Methods Mol. Biol.* 775 311–326. 10.1007/978-1-61779-237-3_17 21863451

[B15] Johnson J. W. Bruckner P. L. Morey D. D. (1990). Relationships among flag leaf characteristics and yield of wheat. *Cereal Res. Commun.* 18 283–289.

[B16] Kirk R. E. (2014). *Experimental Design: Procedures for the Behavioral Sciences*, 4 Edn. California: R Brooks.

[B17] Kromdijk J. Głowacka K. Leonelli L. Gabilly S. T. Iwai M. Niyogi K. K. (2016). Improving photosynthesis and crop productivity by accelerating recovery from photoprotection. *Science* 354 857–861. 10.1126/science.aai8878 27856901

[B18] Li H. Yang Y. Zhang H. Chu S. Zhang X. Yin D. (2016). A Genetic Relationship between Phosphorus Efficiency and Photosynthetic Traits in Soybean As Revealed by QTL Analysis Using a High-Density Genetic Map. *Front. Plant Sci.* 7:924. 10.3389/fpls.2016.00924 PMC4923142 27446154

[B19] Li L. Yang H. Liu P. Ren W. Wu X. Huang F. (2018). Combined impact of heat stress and phosphate deficiency on growth and photochemical activity of sheepgrass (Leymus chinensis). *J. Plant Physiol.* 231 271–276. 10.1016/j.jplph.2018.10.008 30336401

[B20] Li X.-B. Gu J.-D. Zhou Q.-H. (2015). Review of aerobic glycolysis and its key enzymes - new targets for lung cancer therapy. *Thorac. Cancer* 6 17–24. 10.1111/1759-7714.12148 26273330 PMC4448463

[B21] Lin Z.-H. Chen L.-S. Chen R.-B. Zhang F.-Z. Jiang H.-X. Tang N. (2009). CO2assimilation, ribulose-1,5-bisphosphate carboxylase/oxygenase, carbohydrates and photosynthetic electron transport probed by the JIP-test, of tea leaves in response to phosphorus supply. *BMC Plant Biol.* 9:43. 10.1186/1471-2229-9-43 PMC2685392 19379526

[B22] Ma J. Tu Y. Zhu J. Luo W. Liu H. Li C. (2020). Flag leaf size and posture of bread wheat: genetic dissection. *Theor. Appl. Genet.* 133 297–315. 10.1007/s00122-019-03458-2 31628527

[B23] Manoj-Kumar Swarup A. Patra A. K. Chandrakala J. U. Manjaiah K. M. (2018). Effect of elevated CO_2_ and temperature on phosphorus efficiency of wheat grown in an Inceptisol of subtropical India. *Plant Soil Env.* 58 230–235. 10.17221/749/2011-PSE

[B24] Maxwell K. Johnson G. N. (2000). Chlorophyll fluorescence—a practical guide. *J. Exp. Bot.* 51 659–668. 10.1093/jxb/51.345.659 10938857

[B25] McDonald G. Bovill W. Taylor J. Wheeler R. (2015). Responses to phosphorus among wheat genotypes. *Crop Pasture Sci.* 66 430–444.

[B26] Méndez-Espinoza A. M. Romero-Bravo S. Estrada F. Garriga M. Lobos G. A. Castillo D. (2019). Exploring Agronomic and Physiological Traits Associated With the Differences in Productivity Between Triticale and Bread Wheat in Mediterranean Environments. *Front. Plant Sci.* 10:404. 10.3389/fpls.2019.00404 PMC6460938 31024582

[B27] Monyo J. H. Whittington W. J. (1973). Genotypic differences in flag leaf area and their contribution to grain yield in wheat. *Euphytica* 22 600–606. 10.1007/bf00036661

[B28] Murchie E. H. Lawson T. (2013). Chlorophyll fluorescence analysis: a guide to good practice and understanding some new applications. *J. Exp. Bot.* 64 3983–3998. 10.1093/jxb/ert208 23913954

[B29] Nguyen V. L. (2017). *Efficiency in Genotypic Variation and Mechanisms of Phosphorus Use Wheat.* Ph.D thesis, Australia: Flinders University. Ph.D thesis

[B30] Nguyen V. L. Palmer L. Roessner U. Stangoulis J. (2019). Genotypic Variation in the Root and Shoot Metabolite Profiles of Wheat (Triticum aestivum L.) Indicate Sustained, Preferential Carbon Allocation as a Potential Mechanism in Phosphorus Efficiency. *Front. Plant Sci.* 10:995. 10.3389/fpls.2019.00995 PMC6691131 31447867

[B31] Nguyen V. L. Stangoulis J. (2019). Variation in root system architecture and morphology of two wheat genotypes is a predictor of their tolerance to phosphorus deficiency. *Acta Physiol. Plant.* 41:109.

[B32] Ozturk L. Eker S. Torun B. Cakmak I. (2005). Variation in phosphorus efficiency among 73 bread and durum wheat genotypes grown in a phosphorus-deficient calcareous soil. *Plant Soil* 269 69–80. 10.1007/s11104-004-0469-z

[B33] Peng S. Krieg D. R. Girma F. S. (1991). Leaf photosynthetic rate is correlated with biomass and grain production in grain sorghum lines. *Photosynth. Res.* 28 1–7. 10.1007/BF00027171 24414793

[B34] Rao I. M. Fredeen A. L. Terry N. (1993). Influence of phosphorus limitation on photosynthesis, carbon allocation and partitioning in sugar beet and soybean grown with a short photoperiod. *Plant Physiol. Biochem.* 31 223–231.

[B35] Rao I. M. Terry N. (1989). Leaf phosphate status, photosynthesis, and carbon partitioning in sugar beet. *Plant Physiol.* 90 814–819. 16666882 10.1104/pp.90.3.814 PMC1061805

[B36] Ritchie G. A. (2006). “Chlorophyll fluorescence: What is it and what do the numbers mean?,” in *Riley, L.E.; Dumroese, R.K.; Landis, T.D., tech. coords. 2006. National Proceedings: Forest and Conservation Nursery Associations - 2005. Proc. RMRS-P-43* L. E.DumroeseR. K.LandisT. D.coords. (Grassland: Rocky Mountain Research Station), 34–42.

[B37] Roche D. (2015). Stomatal Conductance Is Essential for Higher Yield Potential of C3 Crops. *Crit. Rev. Plant Sci.* 34 429–453.

[B38] Ruban A. V. (2016). Non-photochemical Chlorophyll Fluorescence Quenching: mechanism and Effectiveness in Protecting Plants from Photodamage. *Plant Physiol.* 170 1903–1916. 26864015 10.1104/pp.15.01935 PMC4825125

[B39] Savvides A. M. Fotopoulos V. (2018). Two inexpensive and non-destructive techniques to correct for smaller-than-gasket leaf area in gas exchange measurements. *Front. Plant Sci.* 9:548. 10.3389/fpls.2018.00548 PMC5928467 29740471

[B40] Shi Q. Pang J. Yong J. W. H. Bai C. Pereira C. G. Song Q. (2020). Phosphorus-fertilisation has differential effects on leaf growth and photosynthetic capacity of Arachis hypogaea L. *Plant Soil* 447 99–116. 10.1007/s11104-019-04041-w

[B41] Singh S. K. Reddy V. R. (2014). Combined effects of phosphorus nutrition and elevated carbon dioxide concentration on chlorophyll fluorescence, photosynthesis, and nutrient efficiency of cotton. *J. Plant Nutrition Soil Sci.* 177 892–902. 10.1002/jpln.201400117

[B42] Sterling T. M. (2005). *Transpiration: Water Movement Through Plants.* New Maxico: New Mexico State University. 34 123–123.

[B43] UCLA (n.d.). *FAQ How Can I Understand A Three-Way Interaction In Anova?* Available online at: https://stats.idre.ucla.edu/other/mult-pkg/faq/general/faqhow-can-i-understand-a-three-way-interaction-in-anova/ (accessed March 26, 2021).

[B44] Vance C. P. Uhde-Stone C. Allan D. L. (2003). Phosphorus acquisition and use: critical adaptations by plants for securing a non-renewable resource. *New Phytolo.* 157 423–447. 10.1046/j.1469-8137.2003.00695.x 33873400

[B45] Veronica N. Subrahmanyam D. Vishnu Kiran T. Yugandhar P. Bhadana V. P. Padma V. (2017). Influence of low phosphorus concentration on leaf photosynthetic characteristics and antioxidant response of rice genotypes. *Photosynthetica* 55 285–293.

[B46] Wang J. Song K. Sun L. Qin Q. Sun Y. Pan J. (2019). Morphological and Transcriptome Analysis of Wheat Seedlings Response to Low Nitrogen Stress. *Plants* 8:98. 10.3390/plants8040098 30991719 PMC6524375

[B47] Wasaya A. Manzoor S. Yasir T. A. Sarwar N. Mubeen K. Ismail I. A. (2021). Evaluation of Fourteen Bread Wheat (Triticum aestivum L.) Genotypes by Observing Gas Exchange Parameters. *Sustainability* 13:4799. 10.3390/su13094799

[B48] Xu H. X. Weng X. Y. Yang Y. (2007). Effect of phosphorus deficiency on the photosynthetic characteristics of rice plants. *Russ. J. Plant Physiol.* 54 741–748.

[B49] Xu Q. Ma X. Lv T. Bai M. Wang Z. Niu J. (2020). Effects of Water Stress on Fluorescence Parameters and Photosynthetic Characteristics of Drip Irrigation in Rice. *Water* 12:289.

[B50] Yadav A. K. Carroll A. J. Estavillo G. M. Rebetzke G. J. Pogson B. J. (2019). Wheat drought tolerance in the field is predicted by amino acid responses to glasshouse-imposed drought. *J. Exp. Bot.* 70 4931–4948. 10.1093/jxb/erz224 31189018 PMC6760313

[B51] Yu M. Chen H. Mao S.-L. Dong K.-M. Hou D.-B. Chen G.-Y. (2020). Contribution of photosynthetic- and yield-related traits towards grain yield in wheat at the individual quantitative trait locus level. *Biotechnol. Biotechnol. Equipment* 34 1188–1197.

[B52] Zhang P. Zhang Z. Li B. Zhang H. Hu J. Zhao J. (2020). Photosynthetic rate prediction model of newborn leaves verified by core fluorescence parameters. *Scientific Rep.* 10:3013. 10.1038/s41598-020-59741-6 32080238 PMC7033164

