# Supplementary material for: Higher Photochemical Quenching and Better Maintenance of Carbon Dioxide Fixation Are Key Traits for Phosphorus Use Efficiency in the Wheat Breeding Line, RAC875
Source: Front Plant Sci. 2022 Feb 4;12:816211. doi: 10.3389/fpls.2021.816211 (PMC8854500; doi:10.3389/fpls.2021.816211)
Supplement: Supplementary file 1 [file Data_Sheet_1.docx]

Supplementary Material

Higher photochemical quenching and better maintenance of CO_2_ fixation are key traits for PUE in the wheat breeding line, RAC875.

Van Lam Nguyen^1^, Lachlan Palmer^1^ and James Stangoulis^1*^

^1^College of Science and Engineering, Flinders University, Sturt Rd, Bedford Park SA 5042, Australia.

**^*^Correspondence:**

James Stangoulis

Email: james.stangoulis@flinders.edu.au

Postal address: GPO Box 2100, Adelaide 5001, South Australia

# Supplementary figures


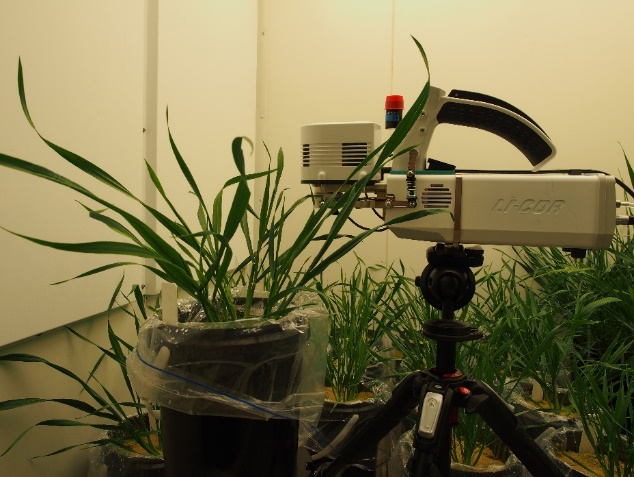


**Figure 1 S.** Gas exchange measurement by LI-6800 portable photosynthesis system

**
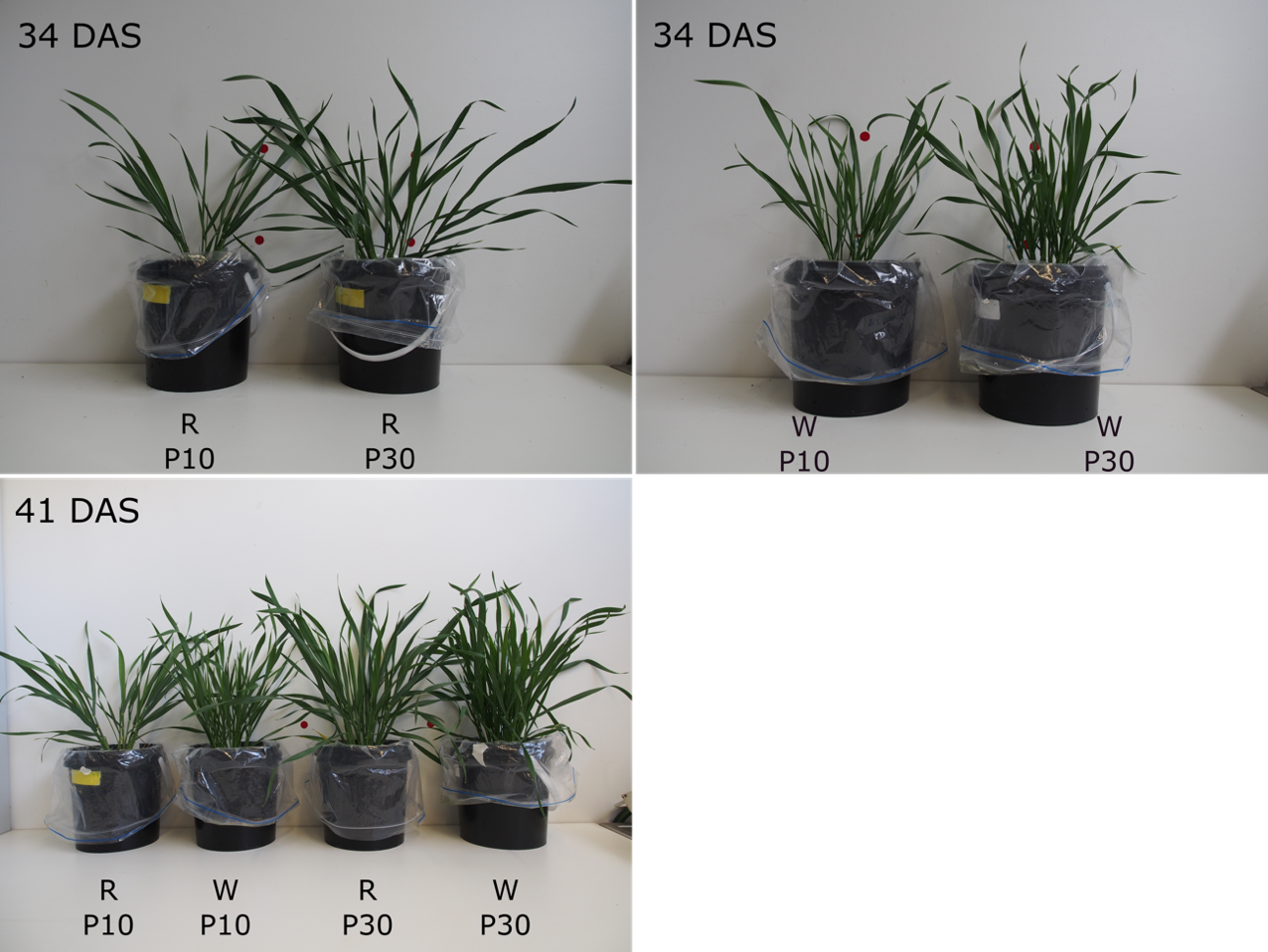
**

**Figure 2 S**. Plant growth at the vegetative stage (34 and 41 DAS). R: RAC875, W: Wyalkatchem, P10 and P30: P supply at 10 and 30 mg P/kgsoil.


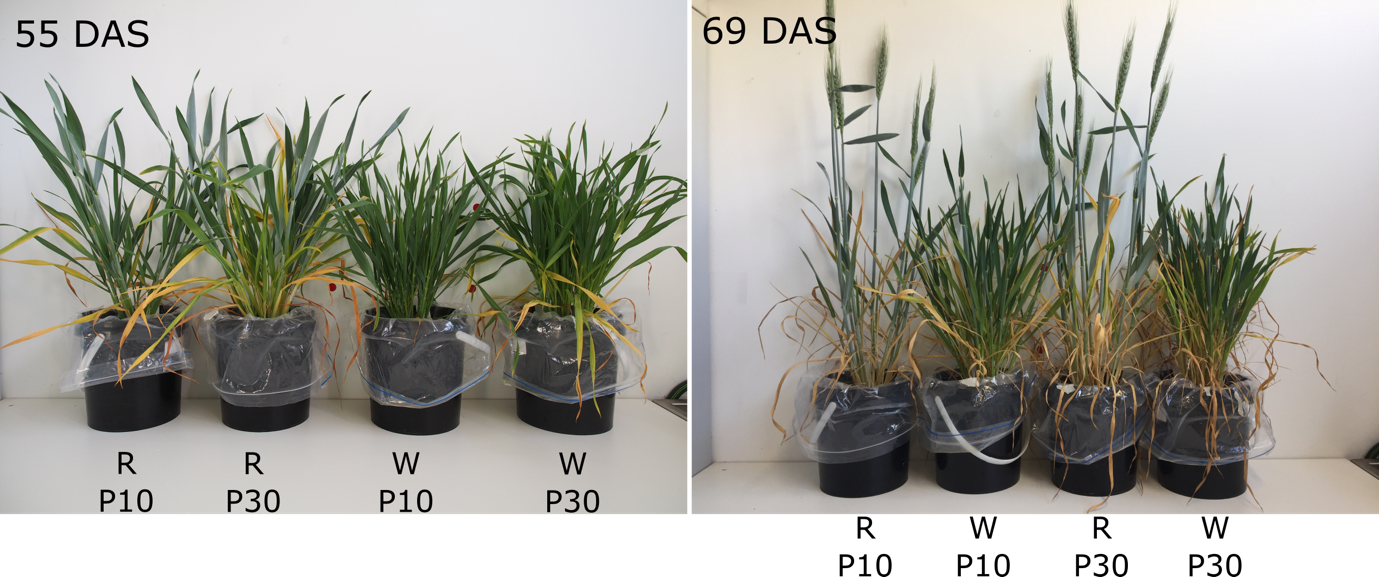


**Figure 3 S**. Plant growth at the booting stage (55 DAS for RAC875 and 69 DAS for Wyalkatchem). R: RAC875, W: Wyalkatchem, P10 and P30: P supply at 10 and 30 mg P/kgsoil.

# Supplementary Tables

**Table 1 S.**Analysis of variance, simple interaction and simple effect for effects of genotype, P supply and growth stage on tiller number. P10 and P30: P supply at 10 mg and 30 mg P/kg soil; DAS: day after sowing.

| **Source** | **SS** | **df** | **MS** | **F** | **Sig** |
| --- | --- | --- | --- | --- | --- |
| Genotype (G) | 1177 | 1 | 1177 | 524.976 | <0.001 |
| P supply (P) | 383 | 1 | 383 | 170.713 | <0.001 |
| Growth stage (GS) | 8712 | 6 | 1452 | 647.918 | <0.001 |
| G x P | 21 | 1 | 21 | 9.566 | 0.0027 |
| G x GS | 1406 | 6 | 234.3 | 104.558 | <0.001 |
| P x GS | 433 | 6 | 72.2 | 32.228 | <0.001 |
| G x P x GS | 32 | 6 | 5.3 | 2.361 | 0.0037 |
| ***G x P at each GS*** |  |  |  |  |  |
| G x P at GS = 13 DAS | 0.25 | 1 | 0.25 | 0.11 | ns |
| G x P at GS = 23 DAS | 3.06 | 1 | 3.06 | 1.39 | ns |
| G x P at GS = 25 DAS | 1.563 | 1 | 1.563 | 0.71 | ns |
| G x P at GS = 27 DAS | 9 | 1 | 9 | 4.09 | ns |
| G x P at GS = 30 DAS | 0.25 | 1 | 0.25 | 0.11 | ns |
| G x P at GS = 34 DAS | 0.25 | 1 | 0.25 | 0.11 | ns |
| G x P at GS = 51 DAS | 36 | 1 | 36 | 16.36 | sig |
| ***G at each GS*** |  |  |  |  |  |
| G at GS = 13 DAS | 4 | 1 | 4 | 1.82 | ns |
| G at GS = 23 DAS | 33.06 | 1 | 33.06 | 15.03 | sig († ‡) |
| G at GS = 25 DAS | 27.562 | 1 | 27.562 | 12.53 | sig († ‡) |
| G at GS = 27 DAS | 36 | 1 | 36 | 16.36 | s sig († ‡) |
| G at GS = 30 DAS | 144 | 1 | 144 | 65.45 | sig († ‡) |
| G at GS = 34 DAS | 175.56 | 1 | 175.56 | 79.8 | sig († ‡) |
| G at GS = 51 DAS | 2162.2 | 1 | 2162.2 | 982.82 | sig († ‡) |
| ***P at each GS*** |  |  |  |  |  |
| P at GS = 13 DAS | 0.25 | 1 | 0.25 | 0.11 | ns |
| P at GS = 23 DAS | 14.06 | 1 | 14.06 | 6.39 | sig (‡) |
| P at GS = 25 DAS | 3.062 | 1 | 3.062 | 1.39 | ns |
| P at GS = 27 DAS | 6.25 | 1 | 6.25 | 2.84 | ns |
| P at GS = 30 DAS | 72.25 | 1 | 72.25 | 32.84 | sig († ‡) |
| P at GS = 34 DAS | 95.06 | 1 | 95.06 | 43.21 | sig († ‡) |
| P at GS = 51 DAS | 625 | 1 | 625 | 284.09 | sig († ‡) |
| ***G at P (GS=51 DAS)*** |  |  |  |  |  |
| G at P10 (GS=51 DAS) | 820.1 | 1 | 820.1 | 372.77 | sig († ‡) |
| G at P30 (GS=51 DAS) | 1378.1 | 1 | 1378.1 | 626.41 | sig († ‡) |
| Error | 188 | 84 | 2.2 |  |  |

^†^F_(0.05/6, 1, 84)_ = 7.30 (Family error rate critical value)

^‡^F_(0.35/10, 1, 84)_ = 4.59 (Dunn’s critical value)

**Table 2 S.** Two-way ANOVA and simple effect analysis for the effects of genotype, P supply on the productive tiller number and the ratio of productive tillers to the total tiller number, leaf number per main tiller at 69 DAS, and the grain yield. Simple effect was analysed using Phia package in r. P10 and P30: P supply at 10 mg and 30 mg P/kg soil.

| **Source** | **P value** |  |  |  |
| --- | --- | --- | --- | --- |
|  | Productive tiller number | Productive tiller/total tiller | Leaf number/mail tiller | Grain yield |
| G (Genotype) | P<0.001 | P<0.001 | P<0.001 | P<0.001 |
| P (P supply) | P<0.001 | P<0.002 | P<0.001 | P<0.001 |
| G x P | P=0.474 | P=0.244 | P=0.346 | P=0.346 |
| ***G at each P supply*** |  |  |  |  |
| G at P10 | P<0.001 | P=0.002 | P<0.001 | P=0029 |
| G at P30 | P<0.001 | P=0.021 | P<0.001 | P=0.276 |

**Table 3 S.**Analysis of variance, simple interaction and simple effect for effects of genotype, P supply and growth stage on intracellular CO_2_concentration at the vegetative stage. P10 and P30: P supply at 10 mg and 30 mg P/kg soil; DAS: day after sowing.

| **Source** | **SS** | **df** | **MS** | **F** | **Sig** |
| --- | --- | --- | --- | --- | --- |
| Genotype (G) | 608.2 | 1 | 608.2 | 7.116 | 0.014 |
| P supply (P) | 44 | 1 | 44 | 0.515 | 0.48 |
| Growth stage (GS) | 0 | 1 | 0 | 0 | 0.982 |
| G x P | 253.7 | 1 | 253.7 | 2.968 | 0.092 |
| G x GS | 471.2 | 1 | 471.2 | 5.513 | 0.028 |
| P x GS | 3.7 | 1 | 3.7 | 0.043 | 0.836 |
| G x P x GS | 2.4 | 1 | 2.4 | 0.028 | 0.87 |
| G x P at each GS |  |  |  |  |  |
| G x P at GS=34 DAS | 108.1 | 1 | 108.1 | 1.26 |  |
| G x P at GS=41 DAS | 157.9 | 1 | 157.9 | 1.85 |  |
| G at each GS |  |  |  |  |  |
| G at GS=34 DAS | 4.4 | 1 | 4.4 | 0.05 | ns |
| G at GS=41 DAS | 1075.1 | 1 | 1075.1 | 12.58 | sig († ‡) |
| Residual | 2051.4 | 24 | 85.475 |  |  |

^†^F_(0.05/2, 1, 24)_ = 5.72 (Family error rate critical value)

^‡^F_(0.35/6, 1, 24)_ = 3.95 (Dunn’s critical value)

**Table 4 S.** The ratio of photosynthetic rate at low P to adequate P for each genotype at the vegetative and booting stage. P10 (low P) and P30 (adequate P): P supply at 10 mg and 30 mg P/kg soil.

| **Genotype** | **Pn** |  |
| --- | --- | --- |
|  | Vegetative | Booting |
| RAC875 | 0.83 | 0.90 |
| Wyalkatchem | 0.89 | 0.83 |

**Table 5 S.** Two-way ANOVA and simple effect analysis for the effects of genotype, P supply on F_o_, F_m_ and F_v_ at booting stage. Simple effect was analysed using Phia package in r.

| **Source** | **P value** |  |  |
| --- | --- | --- | --- |
|  | F_o_ | F_m_ | F_v_ |
| Genotype (G) | P<0.001 | P<0.001 | P<0.001 |
| P supply (P) | P=0.246 | P=0.058 | P<0.042 |
| G x P | P=0.010 | P=0.003 | P=0.003 |
| ***P at each genotype*** |  |  |  |
| P at RAC875 | P=0.021 | P=0.003 | P=0.002 |
| P at Wyalkatchem | P=0.219 | P=0.294 | P=0.331 |

**Table 6 S.** Two-way ANOVA and simple effect analysis for the effects of genotype, P supply on NQP at booting stage. Simple effect was analysed using Phia package in r.

| **Source** | **P value** |
| --- | --- |
|  | NPQ |
| Genotype (G) | P=0.287 |
| P supply (P) | P=0.131 |
| G x P | P=0.094 |
| ***P at each genotype*** |  |
| P at RAC875 | P=0.890 |
| P at Wyalkatchem | P=0.063 |

**Table 7 S.** The ratio of NPQ to adequate P for each genotype at vegetative and booting stage. P10 (low P) and P30 (adequate P): P supply at 10 mg and 30 mg P/kg soil.

| **Genotype** | **NPQ** |
| --- | --- |
| RAC875 | 0.97 |
| Wyalkatchem | 1.52 |

**Table 8 S.** Effect of P supply on harvest index (HI) in two wheat genotypes

| P treatment (mg P kg^-1^ soil) |  | Harvest index (%) |
| --- | --- | --- |
| 10 | RAC875 | 36.43 ± 0.62^a^ |
|  | Wyalkatchem | 28.79 ± 1.31^b^ |
| 30 | RAC875 | 34.46 ± 0.09 |
|  | Wyalkatchem | 31.76 ± 2.35 |
| *P value* |  |  |
| Genotype (G) |  | P<0.001 |
| P supply (P) |  | P=0.997 |
| G × P |  | P=0.002 |

Different letters show significantly different between two genotypes within the same P supply (P<0.05).
